# The impact of various post-exercise interventions on the relief of delayed-onset muscle soreness: a randomized controlled trial

**DOI:** 10.3389/fphys.2025.1622377

**Published:** 2025-08-04

**Authors:** Ming Wei, Xin Liu, Siyu Wang

**Affiliations:** Shenyang Sport University, Shenyang, China

**Keywords:** DOMS, recovery strategies, vibration therapy, exercise physiology, CMI

## Abstract

**Objective:**

This study aimed to compare the effectiveness of various recovery interventions in alleviating delayed-onset muscle soreness (DOMS), focusing on the comparative effects of vibration therapy, functional electrical stimulation (FES), static stretching, massage therapy, and cold-water immersion (CWI).

**Methods:**

This randomized controlled trial (RCT) was conducted at the university’s physical training center from September 2024 to October. A total of 30 healthy male university students were recruited and randomly assigned to six groups, with five participants in each group. Healthy students were recruited via public announcements and randomly assigned to 6 groups: massage therapy (Group A), CMI (Group B), vibration therapy (Group C), static stretching (Group D), FES (Group E), and control (Group F). After DOMS was induced in the quadriceps, participants received the designated recovery interventions. Assessments were conducted at baseline, immediately post-exercise, and at 24, 48, and 72 h, including tensiomyography (TMG), pressure pain threshold (PPT), knee joint range of motion (ROM), isokinetic strength (ISOK), and biochemical markers (CK, Ca^2+^, IL-6).

**Results:**

Thirty participants completed the study. Group C demonstrated the greatest improvement in contraction time (Tc) at 72 h (*p* < 0.05) and the most significant reduction in IL-6 levels (*p* < 0.01). Group E significantly enhanced peak concentric power recovery from 24 to 72 h (*p* < 0.001) and ranked second to Group B in early-phase IL-6 regulation (*p* < 0.05). Group D demonstrated a significant main effect on ROM recovery (*F* = 3.41, *p* < 0.05), while Group A most effectively reduced CK levels and stabilized Ca^2+^ homeostasis (*p* < 0.05). All variables showed significant main effects of time (Tc: *η* ^*2*^ = 0.760; Dm: *η* ^*2*^ = 0.824; IL-6: *η* ^*2*^ = 0.854), with interaction effects noted for Dm (*η* ^*2*^ = 0.360) and peak concentric power (*η* ^*2*^ = 0.336).

**Conclusion:**

Vibration therapy effectively enhanced muscle responsiveness by reducing Tc. Massage therapy was most effective in reducing IL-6, CK, and Ca^2+^ levels, alleviating muscle stiffness and soreness, and facilitating tissue repair. FES significantly increased PPT and muscle strength, mitigating DOMS-related pain and functional decline. Static stretching offered notable benefits in enhancing joint ROM, whereas CMI effectively suppressed early inflammatory responses.

## 1 Introduction

DOMS refers to muscle discomfort that typically occurs 24–72 h after engaging in intense or unaccustomed physical activities, particularly eccentric exercise (Hotfiel et al., 2018). Typical symptoms of DOMS include pain during muscle contraction or palpation, stiffness, reduced strength, and limited ROM (Boobphachart et al., 2017; Haksever et al., 2016). Studies have confirmed that exercising under conditions of DOMS significantly increases the risk of muscle injury (Haksever et al., 2016).

The risk of DOMS is influenced by various factors. Eccentric contractions, especially those involving lengthening under load (e.g., downhill running or slow lowering of weights), are particularly prone to cause ultrastructural damage to muscle fibers—such as Z-line streaming and sarcomere disruption—as well as excessive strain on connective tissues, thereby significantly increasing the risk of DOMS (Fridén et al., 1983; Hough, 1900).Training variables are also critical. High-intensity efforts (>60% 1RM) and high-volume sessions can intensify muscle damage, while insufficient recovery time leads to cumulative strain, such as increased sarcolemma permeability and Ca^2+^ dysregulation (Armstrong, 1984; Armstrong, 1990; Gulick and Kimura, 1996; Hamill et al., 1991; Vila-Chã et al., 2023; Yates and Armbruster, 1990). Inexperienced individuals (e.g., beginners or those returning after inactivity) are more susceptible to DOMS due to suboptimal muscle adaptation, poor proprioception, and individual differences in connective tissue elasticity and repair capacity (Hamill et al., 1991; Smith, 1991; Sydney-Smith and Quigley, 1992; Vila-Chã et al., 2023; Zainuddin et al., 2005). Moreover, DOMS itself can impair proprioceptive function—such as reduced muscle spindle sensitivity and joint position sense—which compromises movement control. It can also induce up to a 43.5% decrease in eccentric strength, triggering compensatory loading in adjacent joints or muscle groups, which further elevates the risk of secondary sports injuries (Hody et al., 2019; Orchard et al., 1997). Although DOMS is generally self-limiting, with symptoms typically resolving within 5–7 days, the absence of timely intervention may result in significant complications. Muscle functional impairment can persist beyond 72 h, and eccentric strength loss may exceed 40% (Yates and Armbruster, 1990), potentially compromising daily activity and disrupting structured training plans. Furthermore, compensatory joint overload is more likely to occur, with studies indicating a 31% increase in injury risk under such conditions (Orchard et al., 1997). The time required to resume training may also be extended beyond 72 h, further affecting athletic performance and recovery timelines (Li et al., 2024). In contrast, appropriately timed and targeted interventions can interrupt the pathological cycle of “pain–stiffness–functional loss” and yield critical clinical benefits. Early-phase anti-inflammatory strategies—such as cold-water immersion—have been shown to reduce IL-6 peak levels by 37.5% (Peake et al., 2017), while functional modalities like FES can accelerate strength recovery by up to 15% (Lake, 1992). Directly reducing the risk of secondary musculoskeletal injuries and shortening the rehabilitation window by 50% (Dupuy et al., 2018).

Clinically, modalities such as cryotherapy, vibration therapy, massage therapy, ultrasound, and FES are commonly used to manage DOMS (Nahon et al., 2021). These interventions primarily act by reducing exercise-induced muscle damage and inflammation. Specifically, they help reduce swelling and edema, limit fluid diffusion into muscle interstitial spaces, and foster a more favorable environment for muscle recovery. Additionally, by enhancing blood and lymphatic circulation, these interventions accelerate the clearance of metabolic waste and inflammatory mediators from muscle tissue into the bloodstream, thereby attenuating muscle damage and inflammatory responses associated with exercise (Dupuy et al., 2018; Minett and Duffield, 2014).

However, the optimal strategy for alleviating DOMS remains unclear. Current evidence on DOMS management presents three key limitations. First, many comparative studies adopt heterogeneous outcome measures—often relying on subjective pain scales rather than objective biological markers such as creatine kinase or interleukins. Additionally, the observation windows are typically too short (less than 48 h), making it difficult to determine the temporal efficacy of interventions (Hody et al., 2019). Second, although most therapies are anti-inflammatory in nature, their pathway-specific effects remain poorly quantified, limiting the development of mechanism-driven, precision-based treatment protocols (Hotfiel et al., 2018). Third, current clinical trials tend to focus on single interventions and overlook the potential benefits of combining multiple strategies that act on different physiological pathways to achieve synergistic effects in DOMS relief (Dupuy et al., 2018; Jukic et al., 2020).

This study specifically focused on healthy male university students to minimize confounding effects associated with sex-related variables—such as hormonal fluctuations during the female menstrual cycle, which may influence inflammatory responses and pain perception (Zainuddin et al., 2005). Existing evidence suggests that sex differences may affect the onset and recovery of DOMS through pathways involving muscle fiber composition, hormonal dynamics, and nociceptive sensitivity (Toumi and Best, 2003; Zainuddin et al., 2005). By limiting participants to males, the present study aimed to isolate the intrinsic physiological effects of each intervention with greater clarity. In clinical settings, the selection of DOMS management strategies is influenced by various practical considerations, including resource availability, individual tolerance, and training phase. These interventions are typically regarded as complementary rather than mutually exclusive. Through multi-timepoint assessments and objective outcome measures, this study systematically quantified the differential effects of five commonly used modalities—vibration therapy, FES, massage, static stretching, and CWI.

Therefore, further comparative investigations are warranted to evaluate the efficacy of diverse recovery strategies in reducing DOMS. Continuous monitoring of DOMS progression at various time points post-intervention may offer theoretical insights and practical guidance for optimizing both the selection and timing of recovery protocols.

## 2 Methods

### 2.1 Study design

**FIGURE 1:**
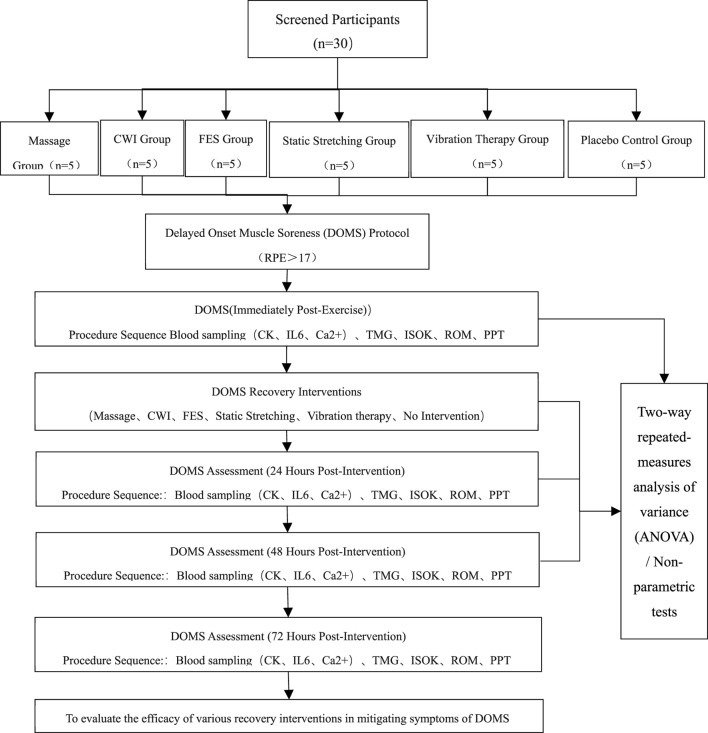
Flowchart.

This randomized, single-blind, controlled trial was conducted at the Physical Training Center of Shenyang Sport University. Outcome assessors were blinded to group allocation using coded labels, while therapists administering the interventions were unavoidably aware of the group assignments. Data were collected between September and October 2024. Participants were randomly allocated to six groups: massage therapy (Group A), CWI (Group B), vibration therapy (Group C), static stretching (Group D), FES, (Group E), and control group (Group F) (Figure 1).

All outcome measurements selected in this study have been widely adopted in previous research and demonstrate acceptable levels of reliability and validity, as supported by the following references, Visual Analogue Scale (VAS), (Beletsky et al., 2020; Chamberlain, 1982), TMG, (Čular et al., 2023), PPT, (Hirschmüller et al., 2017), knee joint ROM, (Chesterton et al., 2007), bilateral quadriceps isokinetic strength, and blood biochemical markers (CK (Fridén et al., 1983), Ca^2+^ (Sydney-Smith and Quigley, 1992), IL-6 (Smith, 1991)). Evaluations were conducted at 24, 48, and72 h post-DOMS induction, starting with the left leg and followed by the right leg. The average value of both legs was used for analysis.

DOMS Induction Protocol. All participants completed a 10-min warm-up, consisting of three sets of 30-s foam rolling exercises followed by 5 minutes of jogging. DOMS was induced in the quadriceps femoris based on a previously established protocol, (Faming and Beixiang, 2017), which included 15 walking deep squat jumps and 30 stationary half-squat jumps with a 10 kg load per set, for a total of 10 sets. A 2-min rest was provided between exercises and sets. The total exercise duration was approximately 50–60 min, effectively inducing DOMS in the quadriceps.

Intervention Protocol. Immediately after DOMS induction, participants received the designated intervention as follows: Group A (Massage Therapy): Manual massage of the quadriceps for 10 min (Guo et al., 2017). Detailed stimulation parameters and session durations for each recovery intervention are summarised in Table 1. Group B (CWI): Submersion in water at 11°C–15°C for 11–15 min (Ferreira-Junior et al., 2014). Group C (Vibration Therapy): Application of a Deep Muscle Stimulator (DMS) to the quadriceps at a frequency of 60 Hz, with 3 min per muscle (Broadbent et al., 2010; Keller et al., 2002). Group D (Static Stretching): Targeted static stretching of the quadriceps, holding each stretch for 1 min while progressively increasing range with controlled breathing (Herbert et al., 2007). Group E (FES): Use of the Compex 5.0 Electrical Stimulator in “Relaxing Massage” mode, with automated intensity adjustment based on muscle status, for approximately 22 min (Broadbent et al., 2010). Group F (Control): No intervention was administered.

**TABLE 1:** Dosage parameters of recovery interventions.

Intervention	Device/Type	Stimulation Parameters	Duration per Session
Deep tissue massage (Chamberlain, 1982)	Deep longitudinal pressure	Pressure: 250–300 kPa; Frequency: 10strokes/min Pain control:VAS 5–6/10	10 min
Vibration therapy (Brock et al., 2004)	DMS	Frequency:60 Hz	10 min
FES (Keller et al., 2002)	Compex^®^ Sport Elite	Pulse width:250 μs; Current:30–45 mA; Duty cycle:5 s on/10 s off	20 min
Cold-water immersion (Sautillet et al., 2021)	Water bucket	Temperature:11°C–15°C; Immersion depth: up to iliac crest	12 min
Static stretching (Behm et al., 2016)	Passive static stretch	Intensity: 80% of ROM; Set interval: 30 s	30 s/muscle group × 3 sets

### 2.2 Participants

Sample size estimation was performed using G Power 3.1 software, with an effect size of 0.33 and a power level of 0.9, resulting in a required total sample size of 30 participants. Thirty healthy male students majoring in sports science were recruited from Shenyang Sport University via posters and online platforms (e.g., We Chat). All participants were aged 18–28 years and were randomly assigned to six groups, with five participants per group. No participants dropped out during the study. The study protocol was reviewed and approved by the Scientific Research Ethics Committee of Shenyang Sport University (Approval No. SYTY20240124) and all participants provided written informed consent prior to enrollment. General characteristics and group assignments are summarized in Table 2.

**TABLE 2:** Baseline characteristics of participants across six groups (mean ± SD).

Group	n	Age (years)	Height (cm)	Weight (kg)
A	5	21.2 ± 1.5	175.4 ± 4.8	68.6 ± 7.2
B	5	20.8 ± 1.3	176.0 ± 5.1	69.4 ± 6.9
C	5	21.5 ± 1.6	174.8 ± 4.3	70.1 ± 8.0
D	5	20.9 ± 1.4	175.9 ± 4.9	67.9 ± 7.5
E	5	21.0 ± 1.7	176.3 ± 5.5	71.2 ± 6.7
F	5	21.3 ± 1.5	175.7 ± 4.7	69.8 ± 7.8
Total	30	21.1 ± 1.4	175.7 ± 4.5	69.5 ± 6.8

Note: Data are presented as mean ± SD. No significant differences between groups (one-way ANOVA): Age: F (5, 24) = 0.15, P = 0.978, Height: F (5, 24) = 0.12, P = 0.988, Weight: F (5, 24) = 0.18, P = 0.967.

Healthy participants were recruited publicly from Shenyang Sport University. All individuals provided written informed consent prior to enrollment, having been fully informed of the study procedures, potential risks, and their rights as participants (Karlsson et al., 2019).

The inclusion criteria were as follows: 1. Healthy male university students; 2. No engagement in regular physical training within the past month; 3. Provided written informed consent prior to participation; 4. Aged between 18 and 28 years; 5. Body mass index (BMI) between 18.5 and 24.0 kg/m^2^; 6. Agreed to abstain from physical activity during the week prior to the trial and throughout the testing period, and to avoid any recovery-related interventions (e.g., medication, supplements, physical therapies, or massage).

Exclusion Criteria: 1. Failure to complete the experimental procedures as instructed. 2. Voluntary withdrawal from the trial at any point; 3. Use of any additional recovery interventions during the experimental period. Eligibility was confirmed via a standardized health screening conducted by a certified rehabilitation therapist. Participants were randomly assigned using a computer-generated random number sequence. A randomization code table was established to ensure allocation concealment and maintain objectivity throughout the group assignment process.

### 2.3 Outcome measurements

#### 2.3.1 Physiological assessments

VAS is widely used in clinical pain assessments and the evaluation of pain-related treatments. Numerous clinical studies have confirmed that VAS is one of the most sensitive and reliable methods for measuring pain intensity. It is easy to understand and apply, and most patients consider a VAS score between 4 and 6 (on a 10-point scale) to be within an acceptable pain range (Beletsky et al., 2020; Chamberlain, 1982).

ROM was assessed using a standard goniometer. Participants were positioned prone with the hips in a neutral position. The assessor passively flexed the participant’s knee until an end-feel was perceived or pelvic movement was observed. Each leg underwent three consecutive trials, and the highest value was recorded for analysis (Hancock et al., 2018).

Muscle mechanical response was evaluated using a TMG system. A single electrical pulse (1 ms duration, 20–80 mA intensity) was applied to stimulate the rectus femoris. Two primary parameters were recorded: Tc—the time required for the muscle to contract from 10% to 90% of its full contraction following stimulation, and Dm—the greatest muscle deformation observed from minimal to maximal stimulation (Čular et al., 2023).

The PPT of three quadriceps sites (vastus medialis, rectus femoris, and vastus lateralis) was quantified using a handheld digital algometer (HP-50, China). Pressure was applied perpendicularly at a rate of 1 kg/s until the participant reported pain. Each site was tested three times with a consent 10-s interval between trials, and the average value was recorded (Chesterton et al., 2007). All assessments were conducted bilaterally, beginning with the unaffected side in a standardized sequence for each participant.

An isokinetic dynamometer (ISOMED 2000; Germany) was used to assess quadriceps peak torque (NM). Participants were seated with their lateral femoral epicondyle aligned with the axis of rotation of the device. The trunk, pelvis, and distal third of the thigh were securely stabilized, and participants were instructed to hold the handles on either side of the seat. Testing was performed at an angular velocity of 240°/s, involving five maximal concentric contractions. The highest peak torque value was retained for analysis (Hirschmüller et al., 2017).

#### 2.3.2 Blood biochemical analysis

At five time points—pre-exercise, immediately post-exercise, and at 24, 48, and72 h post-intervention—3 mL of venous blood was drawn from the antecubital vein by a trained nurse. Blood samples were centrifuged using a tabletop centrifuge (TGL-16c, Shanghai, China) at 3,000 rpm for 15 min to separate the serum. Each serum was immediately aliquoted and stored in liquid nitrogen tanks at −196°C until analysis. Serum concentrations of CK, Ca^2+^, and IL-6 were quantitatively determined using a fully automated biochemical analyzer, according to standardized protocols.

### 2.4 Statistical analysis

All statistical analyses were performed using SPSS version 27.0 (IBM Corp, Armonk, NY, United States). Data were presented as mean ± standard deviation (SD). A two-way repeated-measures analysis of variance (ANOVA) was conducted to evaluate the effects of different recovery interventions and time points on the following outcome variables: Tc, Dm, PPT, peak concentric power, ROM, CK, Ca^2+^, and IL-6.

Baseline comparisons were conducted to assess group comparability after randomization. Demographic and clinical characteristics were compared across groups. The Shapiro–Wilk test was used to assess the normality of continuous variables. Normally distributed variables were analyzed using parametric tests, while non-normally distributed variables were analyzed using the Mann–Whitney U test. Categorical variables were compared using the chi-square test. When the expected frequency of any cell was less than 20% of the total sample, Fisher’s exact test was applied. All data were expressed as mean ± standard deviation (SD), and statistical significance was set at *p* < 0.05. Baseline characteristics that showed significant between-group differences were used as covariates in the main efficacy and safety analyses after randomization (Ghasemi and Zahediasl, 2012; Schulz et al., 2010).

When significant interaction effects were detected, simple effects analyses were performed to further investigate the main effects. The Bonferroni *post hoc* test was applied to compare differences between groups and across time points for all assessed variables. The significance level was adopted at α = 0.05 for all statistical analyses.

## 3 Result

A total of 30 participants were randomly assigned to six groups (*n* = 5 each), and all completed the intervention and follow-up assessments. Baseline demographic characteristics, including age, height, and weight, are summarized in Table 2. There were no significant differences among the groups at baseline (*p* > 0.05), indicating successful randomization.

### 3.1 TC

There were no statistically significant differences in Tc between groups prior to DOMS induction (*p* > 0.05). The Shapiro–Wilk test confirmed that the Tc data in all groups followed a normal distribution. The results of the two-way repeated-measures ANOVA are summarized in Table 3. The interaction effect between time and group was not statistically significant (*F* = 6.404, *p* > 0.05, partial *η* ^*2*^ = 0.550). A significant main effect of time was observed (*F* = 16.642, *p* < 0.001, partial *η* ^*2*^ = 0.760), while the main effect of group was not significant (*F* = 1.615, *p* > 0.*05*, partial *η* ^*2*^ = 0.235).

**TABLE 3:** Tc Assessed by TMG (mean ± SD, n = 30, ms).

Group	Pre-exercise	Post-instant	Post-24 h	Post-48 h	Post-72 h	Main effect of time (F)	Main effect of group (F)	Interaction effect (F)
A	21.82 ± 2.53	29.05 ± 5.08^b^	25.11 ± 6.27	21.65 ± 2.30^c^	21.77 ± 2.34^c^	16.811	16.642	6.404
B	21.72 ± 1.65	26.85 ± 3.50^b^	24.10 ± 4.08	23.50 ± 4.21	22.46 ± 3.06			
C	21.36 ± 2.58	30.38 ± 2.55^b^	23.27 ± 2.44^c^	21.97 ± 2.39^c^	19.35 ± 0.86^c^			
D	20.86 ± 4.84	26.96 ± 5.22^b^	26.61 ± 5.21	25.16 ± 2.56	24.51 ± 1.13			
E	23.85 ± 3.21	28.01 ± 1.45^b^	23.73 ± 5.52	22.94 ± 4.94^c^	24.31 ± 4.99			
F	23.54 ± 2.43	28.12 ± 2.06	24.16 ± 3.65	26.61 ± 2.70	25.62 ± 2.41			
P						0.000	0.743	0.076

Note: A = massage group, B = Cold-water immersion group, C = vibration group, D = stretching group, E = electrical stimulation group, F = control group; the same applies to the following tables. *a* indicates p < 0.05 compared to the control group at the same time point; *aa* indicates p < 0.01 compared to the control group at the same time point; *b* indicates p < 0.05 compared to the pre-exercise value within the same group; *bb* indicates p < 0.01 compared to the pre-exercise value within the same group; *c* indicates p < 0.05 compared to the immediate post-exercise value within the same group; *cc* indicates p < 0.01 compared to the immediate post-exercise value within the same group; *d* indicates p < 0.05 compared to the 24-h post-exercise value within the same group; *dd* indicates p < 0.01 compared to the 24-h post-exercise value within the same group.

Simple effects analysis indicated that the vibration therapy group exhibited a significant difference compared to the control group at 72 h post-exercise (*p* < 0.05). Post-hoc multiple comparisons indicated that the vibration group achieved the most favorable improvement in terms of Tc.

### 3.2 DM

There were no statistically significant differences in Dm between groups prior to DOMS induction (*p* > 0.05). The Shapiro–Wilk test confirmed that the Dm data in all groups were normally distributed. The two-way repeated-measures ANOVA results are presented in Table 4. A statistically significant interaction effect between time and group was observed (*F* = 2.699, *p* < 0.05, partial *η* ^*2*^ = 0.360). A significant main effect of time was also absorbed (*F* = 112.509, *p* < 0.001, partial *η* ^*2*^ = 0.824), whereas the main effect of group was not statistically significant (*F* = 0.833, *p* > 0.05, partial *η* ^*2*^ = 0.148).

**TABLE 4:** Dm Assessed by TMG (mean ± SD, n = 30, mm).

Group	Pre-exercise	Post-instant	Post-24 h	Post-48 h	Post-72 h	Main effect of time (F)	Main effect of group (F)	Interaction effect (F)
A	7.38 ± 0.45	2.49 ± 0.74^bb^	5.38 ± 0.76^bcc^	6.25 ± 0.41^cc^	6.59 ± 0.57^cc^	112.509	0.833	2.669
B	7.59 ± 0.81	2.78 ± 1.20^bb^	3.20 ± 1.08^bb^	3.98 ± 0.41^bb^	4.42 ± 1.00^bbc^			
C	8.56 ± 0.87	3.28 ± 0.80^bb^	4.64 ± 1.60^bbc^	4.46 ± 0.78^bb^	5.54 ± 0.55^bbcc^			
D	7.76 ± 1.50	3.01 ± 2.02^bb^	3.39 ± 2.55^bb^	4.26 ± 2.90^bb^	4.93 ± 2.80^bbc^			
E	8.30 ± 0.57	4.31 ± 2.01^bb^	4.45 ± 1.04^bb^	4.38 ± 0.91^bb^	5.79 ± 1.82^bbc^			
F	8.30 ± 0.98	2.93 ± 0.76^bb^	3.34 ± 1.33^bb^	4.17 ± 1.48^bb^	4.38 ± 1.38^bbc^			
P						0.000	0.539	0.003

Although no significant overall differences were detected between groups, variations in recovery trends were noted across time points. The massage therapy group consistently demonstrated a superior recovery profile among all groups. At each post-exercise time point.

### 3.3 PPT

There were no statistically significant differences in PPT between groups prior to DOMS induction (*p* > 0.05). The Shapiro–Wilk test confirmed that the PPT data in all groups followed a normal distribution. Results of the two-way repeated-measures ANOVA are shown in Table 5. The interaction effect between time and group was not statistically significant (*F* = 1.701, *p* > 0.05, partial *η* ^*2*^ = 0.262). A significant main effect of time was observed (*F* = 21.279, *p* < 0.001, partial *η* ^*2*^ = 0.470), while the main effect of group was not significant (*F* = 1.289, *p* > 0.05, partial *η* ^*2*^ = 0.212).

**TABLE 5:** Changes in PPT (mean ± SD, n = 30, kg).

Group	Pre-exercise	Post-instant	Post-24 h	Post-48 h	Post-72 h	Main effect of time (F)	Main effect of group (F)	Interaction effect (F)
A	7.25 ± 1.024	5.868 ± 1.164^bb^	4.852 ± 1.483^bb^	5.852 ± 1.662^cc^	6.308 ± 1.697^cc^	21.279	1.289	1.701
B	6.064 ± 2.679	4.406 ± 1.169^bb^	4.406 ± 1.947^bb^	4.238 ± 1.791^bb^	4.34 ± 1.217^bb^			
C	6.762 ± 0.904	4.262 ± 0.582^bb^	4.532 ± 0.847^bb^	5.252 ± 1.422^bc^	6.572 ± 2.409^cc^			
D	7.148 ± 2.157	3.932 ± 1.247^bb^	4.992 ± 0.785^bb^	5.67 ± 1.324^bc^	6.094 ± 2.195^cc^			
E	7.462 ± 1.234	4.33 ± 1.179^bb^	6.95 ± 0.921^cc^	7.266 ± 1.003^cc^	7.232 ± 1.118^cc^			
F	6.848 ± 1.745	5.14 ± 1.479^bb^	5.6 ± 1.697^bb^	5.76 ± 2.175^bb^	5.99 ± 2.269^cc^			
P						0.000	0.301	0.086

Although no significant overall differences were found between groups, variations in recovery patterns were observed across time points. FES group consistently demonstrated a favorable recovery trend compared to other groups across all post-exercise time points.

### 3.4 ROM

There were no statistically significant differences in ROM between groups prior to DOMS induction (*p* > 0.05). The Shapiro–Wilk test confirmed that the PPT data in all groups followed a normal distribution. Results of the two-way repeated-measures ANOVA are presented in Table 6. A significant interaction effect between time and group was observed (*F* = 2.444, *p* < 0.05, partial *η* ^*2*^ = 0.337). The main effect of time was not statistically significant (*F* = 1.567, *p* > 0.05, partial *η* ^*2*^ = 0.061), while a significant main effect of group was found (*F* = 3.410, *p* < 0.05, partial *η* ^*2*^ = 0.452).

**TABLE 6:** Changes in ROM (x _ ±s, n = 30, °).

Group	Pre-exercise	Post-instant	Post-24 h	Post-48 h	Post-72 h	Main effect of time (F)	Main effect of group (F)	Interaction effect (F)
A	119.5 ± 5.443	118.9 ± 7.749^bb^	114.9 ± 4.336^bb^	120.6 ± 3.56^cc^	118.7 ± 8.296^bb^	1.567	3.41	0.337
B	120.3 ± 7.653	123 ± 6.413^bb^	118.8 ± 10.305^bb^	120.3 ± 8.765^bb^	119.1 ± 10.52^bb^			
C	121.6 ± 5.055	124.6 ± 4.219^bb^	124.2 ± 6.916^bb^	123.9 ± 3.362^bb^	121.9 ± 8.104^bb^			
D	128.2 ± 2.864	110.3 ± 4.438^bb^	126.7 ± 2.775^cc^	127.3 ± 2.774^bb^	127.3 ± 2.729^bb^			
E	125.7 ± 6.34	121.85 ± 7.512^bb^	122.3 ± 3.752^bb^	123.2 ± 6.419^bb^	123.1 ± 3.38^bb^			
F	125.3 ± 4.04	122.6 ± 6.279^bb^	125.6 ± 4.814^bb^	125.8 ± 7.604^bb^	124 ± 4.937^bb^			
P						0.198	0.018	0.004

Although no significant differences were observed across time points within groups, differences between groups at the same time points were evident. The static stretching group consistently demonstrated superior values in ROM values compared to other groups across all post-intervention time points.

### 3.5 Peak concentric power

There were no statistically significant differences in peak concentric power between groups prior to DOMS induction (*p* > 0.05). The Shapiro–Wilk test confirmed that the peak concentric power data in all groups followed a normal distribution. Results of the two-way repeated-measures ANOVA are presented in Table 7. A statistically significant interaction effect between time and group was observed (*F* = 2.434, *p* < 0.05, partial *η* ^*2*^ = 0.336). Both the main effect of time (*F* = 30.737, *p* < 0.001, partial *η* ^*2*^ = 0.562) and the main effect of group (*F* = 2.915, *p* < 0.001, partial *η* ^*2*^ = 0.378) were statistically significant.

**TABLE 7:** Changes in Peak Concentric Power (mean ± SD, n = 30, W/ms).

Group	Pre-exercise	Post-instant	Post-24 h	Post-48 h	Post-72 h	Main effect of time (F)	Main effect of group (F)	Interaction effect (F)
A	196.68 ± 10.93	132.42 ± 20.89^b^	161.86 ± 18.29^bbc^	170.72 ± 15.00^abbc^	183.03 ± 10.46^bbccd^	30.737	2.915	2.434
B	183.87 ± 8.54	131.71 ± 19.86^b^	161.85 ± 6.01^bc^	167.05 ± 8.75^abc^	173.92 ± 16.45^c^			
C	187.71 ± 9.42	133.60 ± 18.33^bb^	162.03 ± 15.46^bc^	169.09 ± 7.68^abc^	169.88 ± 18.15^c^			
D	188.23 ± 11.66	131.51 ± 22.05^bb^	166.17 ± 7.67^abc^	170.60 ± 10.10^abc^	163.43 ± 14.97^bc^			
E	197.85 ± 12.03	139.65 ± 23.30^b^	189.09 ± 12.16^acc^	191.81 ± 11.24^acc^	197.45 ± 21.60^acc^			
F	192.97 ± 9.23	119.50 ± 36.84^bb^	130.35 ± 27.56^bb^	134.94 ± 20.01^bb^	153.46 ± 19.49^bbcd^			
P						0.000	0.034	0.01

Simple effects analysis revealed that both the static stretching group and the FES group showed significantly greater peak concentric power than the control group at 24 h post-exercise (*p* < 0.05). Post-hoc comparisons indicated that the FES group demonstrated the most favorable recovery. At 48 h post-exercise, all intervention groups (massage therapy, cold water immersion, vibration therapy, static stretching, and FES) exhibited significant differences compared to the control group (*p* < 0.05), with the FES group again showing the greatest enhancement. Furthermore, at 72 h post-exercise, the FES group maintained a significant difference compared to the control group, consistently demonstrating sustained recovery benefits.

### 3.6 IL-6

There were no statistically significant differences in IL-6 levels between groups prior to DOMS induction (p > 0.05). The Shapiro–Wilk test confirmed that the IL-6 data across all groups followed a normal distribution. Results of the two-way repeated-measures ANOVA are presented in Table 8. A statistically significant interaction effect between time and group was observed (*F* = 4.298, *p* < 0.01, partial *η* ^*2*^ = 0.472). Both the main effect of time (*F* = 140.088, *p* < 0.01, partial *η* ^*2*^ = 0.854) and the main effect of group (*F* = 4.255, *p* < 0.01, partial *η* ^*2*^ = 0.733) were statistically significant.

**TABLE 8:** Changes in Interleukin-6 (IL-6) Levels (mean ± SD, n = 30, pg/mL).

Group	Pre-exercise	Post-instant	Post-24 h	Post-48 h	Post-72 h	Main effect of time (F)	Main effect of group (F)	Interaction effect (F)
A	165.53 ± 17.21	272.81 ± 28.70^bb^	229.09 ± 29.30^ab^	187.01 ± 13.12^ac^	169.15 ± 9.02^aacc^	140.088	13.167	4.298
B	166.75 ± 13.06	284.56 ± 20.26^bb^	184.70 ± 31.42^aacc^	148.85 ± 22.13^aacc^	166.06 ± 7.31^aacc^			
C	172.54 ± 19.13	285.58 ± 40.81^bb^	239.87 ± 34.78^abc^	198.56 ± 10.63^c^	159.68 ± 3.04^aaccdd^			
D	161.85 ± 10.24	292.85 ± 21.01^bb^	239.28 ± 27.64^abbc^	193.19 ± 23.81^acc^	176.36 ± 15.70^ccd^			
E	169.48 ± 7.09	280.22 ± 33.07^bb^	190.51 ± 18.89^aacc^	192.10 ± 24.04^ac^	177.00 ± 9.39^cc^			
F	168.49 ± 15.77	285.08 ± 20.93^bb^	308.26 ± 30.87^bb^	238.36 ± 24.45^bbd^	192.75 ± 10.46^ccdd^			
P						0.000	0.000	0.000

At 24 h post-exercise,simple effects analysis revealed that, the massage therapy, cold water immersion, vibration therapy, static stretching, and FES groups all showed significant differences compared to the control group (*p* < 0.05), with the CWI group demonstrating the most effective reduction in IL-6 levels, followed by the FES group.

At 48 h post-exercise, significant differences were observed for the massage, CWI, static stretching, and FES groups compared to the control group, with the CWI group again demonstrating the most effective anti-inflammatory response.

By 72 h post-exercise, the massage, CWI, and vibration therapy groups exhibited significant differences compared to the control group, with the vibration therapy group demonstrating the most favorable IL-6 reduction at this time point.

### 3.7 Ca^2+^

There were no statistically significant differences in Ca^2+^ levels between groups prior to DOMS induction (*p* > 0.05). The Shapiro–Wilk test confirmed that the Ca^2+^ data across all groups followed a normal distribution. Results of the two-way repeated-measures ANOVA are presented in Table 9. The interaction effect between time and group was not statistically significant (*F* = 1.785, *p* > 0.05, partial *η* ^*2*^ = 0.271). A statistically significant main effect of time was observed (*F* = 35.673, *p* < 0.001, partial *η* ^*2*^ = 0.598), whereas the main effect of group was not significant (*F* = 1.571, *p* > 0.05, partial *η* ^*2*^ = 0.247).

**TABLE 9:** Changes in Ca^2+^ Levels (mean ± SD, n = 30, mmol/L).

Group	Pre-exercise	Post-instant	Post-24 h	Post-48 h	Post-72 h	Main effect of time (F)	Main effect of group (F)	Interaction effect (F)
A	3.778 ± 0.367	7.153 ± 0.919^b^	5.711 ± 0.804^ab^	5.001 ± 0.616^ab^	4.107 ± 0.417^ab^	35.673	1.571	1.785
B	3.898 ± 1.341	6.154 ± 0.806^b^	7.566 ± 2.262^b^	6.933 ± 2.18^a^	5.481 ± 1.146^ab^			
C	3.167 ± 1.228	7.131 ± 3.532^b^	8.315 ± 3.068^a^	7.15 ± 1.311^ab^	6.283 ± 1.023^c^			
D	3.992 ± 0.463	8.107 ± 1.16^b^	7.099 ± 2.73^ab^	7.194 ± 2.01^ab^	6.188 ± 1.096^cd^			
E	3.133 ± 0.342	7.25 ± 2.303^b^	7.162 ± 2.549^ab^	6.586 ± 1.853^ab^	5.395 ± 1.35^cd^			
F	6.321 ± 1.516	7.834 ± 1.329^b^	6.538 ± 1.621^b^	6.453 ± 1.11^c^	5.172 ± 2.448^d^			
P						0.000	0.206	0.074

Although no statistically significant overall differences were detected between groups, variations in recovery trends were observed across time points. The massage therapy group consistently demonstrated more effective regulation of Ca^2+^ levels at each post-exercise time point compared to other groups.

### 3.8 CK

There were no statistically significant differences in Ca^2+^ levels between groups prior to DOMS induction (*p* > 0.05). The Shapiro–Wilk test confirmed that the CK data across all groups followed a normal distribution. Results of the two-way repeated-measures ANOVA are presented in Table 10. The interaction effect between time and group was not statistically significant (*F* = 1.536, *p* > 0.05, partial *η* ^*2*^ = 0.242). However, both the main effect of time (*F* = 84.907, *p* < 0.01, partial *η* ^*2*^ = 0.780) and the main effect of group (*F* = 5.750, *p* < 0.05, partial *η* ^*2*^ = 0.545) were statistically significant.

**TABLE 10:** Changes in CK Levels (mean ± SD, n = 30, U/L).

Group	Pre-exercise	Post-instant	Post-24 h	Post-48 h	Post-72 h	Main effect of time (F)	Main effect of group (F)	Interaction effect (F)
A	143.31 ± 15.01	245.73 ± 35.18^b^	192.73 ± 22.87^a^	168.83 ± 15.94^ab^	132.06 ± 11.01^ab^	84.907	5.750	1.536
B	139.48 ± 16.77	305.85 ± 51.98^bb^	231.71 ± 30.20^bb^	189.22 ± 8.29^abbc^	154.53 ± 30.91^ccd^			
C	147.94 ± 13.62	248.24 ± 55.30^b^	201.18 ± 19.94^a^	182.96 ± 13.49^ab^	145.53 ± 16.32^c^			
D	138.75 ± 12.31	278.51 ± 53.72^b^	227.04 ± 27.36^b^	184.47 ± 18.48^abc^	159.59 ± 15.35^cd^			
E	149.33 ± 14.41	329.16 ± 78.02^bb^	223.20 ± 34.09^bc^	177.02 ± 11.74^acc^	148.45 ± 18.91^ccd^			
F	137.95 ± 14.03	274.04 ± 65.70^b^	276.88 ± 66.05^bb^	231.18 ± 30.22^bb^	181.22 ± 10.78^bcd^			
P						0.000	0.001	0.150

At 24 h post-exercise, simple effects analysis showed that both the massage therapy group and the static stretching group exhibited statistically significant differences compared to the control group (*p* < 0.05), with the massage group demonstrating the most favorable recovery.

At 48 h post-exercise, significant differences were observed in all intervention groups (massage, CWI, vibration therapy, static stretching, and FES) compared to the control group, with the massage group showing the best recovery, followed by the FES group.

At 72 h post-exercise, only the massage group maintained a significant difference compared to the control group, continuing to demonstrate the most effective reduction in CK levels.

## 4 Discussion

### 4.1 Effects of different interventions on physiological responses associated with DOMS

This study aimed to compare the effectiveness of various recovery interventions in alleviating DOMS, with a particular focus on vibration therapy, FES, static stretching, massage therapy, and CWI. The results showed that vibration therapy was the most effective in improving muscle reaction latency and reducing Tc; static stretching was the preferred method for enhancing knee joint ROM; FES performed best in improving muscle strength and relieving PPT; cold-water immersion demonstrated advantages in suppressing early-stage inflammatory responses; and massage therapy proved most effective in reducing inflammation, relieving muscle stiffness and soreness, and promoting tissue repair. In this study, although the overall group effect and interaction effect for contraction time (Tc) were not significant, the vibration therapy group exhibited significantly greater recovery at 72 h post-exercise compared to the control group (*p* < 0.05). Vibration therapy facilitates the activation of spinal reflex pathways, including tendon reflexes and muscle spindle reflexes, and preferentially recruits Type II muscle fibers, thereby significantly shortening Tc and improving delayed muscle responsiveness associated with DOMS (Arora et al., 2021; Chang et al., 2022; Ishikura, 2024; Lu et al., 2021). This finding is consistent with that of Chang et al. (2022), who reported that vibration therapy enhances the recruitment of type II muscle fibers, (Chang et al., 2022), However, the Tc improvement observed in the present study was even more pronounced, likely due to optimized intervention parameters (e.g., frequency = 60 Hz, duration = 10 min) and the selection of outcome assessment timing (i.e., the 72-h post-exercise recovery window as a critical phase).

In contrast, massage therapy showed limited efficacy in improving Tc, primarily due to its focus on modulating peripheral tissue compliance rather than neuromuscular activation pathways (Davis et al., 2020; Herrera and Osorio-Fuentealba, 2024; Seo et al., 2021; Weerapong et al., 2005). This result supports the viewpoint proposed by Weerapong et al. (2005), who argued that massage therapy has limited capacity to improve neuromuscular activation pathways, such as those influencing Tc, (Weerapong et al., 2005), However, the present study observed a more pronounced effect of massage therapy on Dm compared to previous studies (Seo et al., 2021). This enhancement may be attributed to the use of deep tissue massage techniques, which are particularly effective in regulating fascial tension and tissue viscoelasticity, thereby optimizing the mechanical properties associated with Dm. However, results for Dm revealed a significant interaction effect between time and group (*p* < 0.05). Massage therapy proved particularly effective in alleviating muscle stiffness and tension induced by DOMS. Its mechanisms include enhancing local blood flow, reducing sympathetic nervous system tone, and promoting fascial remodeling, all of which contribute to relieve muscle swelling and stiffness (Miroshnychenko et al., 2017; Seo et al., 2021; Zainuddin et al., 2005). By comparison, although cold water immersion effectively suppresses acute inflammatory responses, it may also lead to increased muscle stiffness and impaired ion exchange, potentially causing stagnation in Dm recovery and delaying the resolution of DOMS symptoms (Buoite et al., 2024; Xiao et al., 2023). This aligns with the findings of Xiao et al. (2023), who suggested that cold-water immersion may hinder long-term recovery (Xiao et al., 2023), Notably, this study is the first to provide direct experimental evidence for this mechanism using the objective biomechanical marker Dm, showing that elevated muscle stiffness and impaired ion exchange may be the key pathophysiological contributors to delayed Dm recovery in the context of cold-water immersion (Buoite et al., 2024; Xiao et al., 2023).

Regarding the PPT, although the overall group effect did not reach statistical significance, the FES group consistently demonstrated superior improvement across all time points, with a sustained upward trend particularly evident at 48 and 72 h post-exercise. FES “has been shown to effectively increase PPT and alleviate muscle tenderness associated with DOMS through multiple mechanisms.

Primarily, FES activates spinal-level gate control” mechanisms, inhibiting the transmission of nociceptive signals through small-diameter pain fibers, thereby reducing pain perception. This analgesic mechanism strongly supports the applicability of Ohga Set al.'s (2024) theoretical model to the field of DOMS (Ohga et al., 2024). Furthermore, this study revealed that, compared to traditional transcutaneous electrical nerve stimulation (TENS), FES exhibits a more sustained effect in increasing PPT in individuals with DOMS.

This breakthrough finding is attributed to the innovative FES parameter settings used in this study, such as pulse frequency and width specifically targeting motor nerves. These parameters enabled the simultaneous activation of large-diameter afferents and recruitment of deep motor units, thereby amplifying the inhibitory effect on pain pathways. Additionally, FES promotes the release of endogenous opioids, such as β-endorphins, thereby enhancing central analgesic effects. It also stimulates descending inhibitory pathways, facilitating the release of serotonin (5-HT) and norepinephrine, both of which further suppress pain transmission at the spinal level.

Moreover, studies have shown that FES can upregulate the release of brain-derived neurotrophic factor (BDNF) and anti-inflammatory cytokines such as interleukin-10 (IL-10), thereby contributing to a synergistic analgesic effect involving both peripheral and central mechanisms (Bryant et al., 2023; Ohga et al., 2024; Rudolph et al., 2023).

In contrast, although cold water immersion is effective in suppressing early-stage inflammation, its analgesic efficacy remains limited. The associated reduction in blood flow may even delay PPT recovery by impairing metabolic clearance and prolonging tissue stiffness (Pinto et al., 2020; Vinck et al., 2006; Xiao et al., 2023).

These results challenge the consensus proposed by Pinto et al. (2020), which emphasized the general benefits of cold-water immersion in managing DOMS (Pinto et al., 2020). For the first time, this study demonstrated through dynamic changes in PPT that CWI-induced suppression of local blood flow—exceeding a 40% reduction—may impair the clearance of metabolic waste and contribute to the accumulation of tissue stiffness, thereby significantly diminishing its analgesic efficacy. This mechanistic finding clearly indicates that the clinical application of CWI must involve careful consideration of its short-term anti-inflammatory benefits versus its potential long-term drawbacks in pain management. Regarding ROM, the static stretching group demonstrated a consistent advantage at all intervention time points, with a statistically significant group effect observed (*p* < 0.05). Static stretching effectively improves ROM by decreasing muscle spindle sensitivity and relieving central inhibition, making it the preferred strategy for alleviating joint stiffness associated with DOMS (Behm et al., 2016; Konrad et al., 2024).

This study not only validated the core mechanism proposed by Konrad et al. (2024)—that stretching improves range of motion (ROM) by reducing muscle spindle sensitivity and alleviating central inhibition (Konrad et al., 2024)—but also extended these findings by revealing a sustained clinical benefit of static stretching under the pathological context of DOMS, characterized by a vicious cycle of pain and stiffness. Specifically, the effect of static stretching in relieving joint stiffness remained consistently significant throughout the critical recovery window from 24 to 72 h post-intervention (*p* < 0.05). This is the first study to provide longitudinal evidence supporting static stretching as a long-term joint function management strategy for DOMS.

Microdamage and impaired neural drive associated with DOMS have been shown to significantly reduce muscle strength and power output (Lau and Nosaka, 2011). In this study, the FES group demonstrated significantly greater recovery in peak concentric power compared to the control group at 24, 48, and 72 h post-exercise (*p* < 0.05), indicating the most effective intervention among all groups. These findings suggest that FES can counteract DOMS-induced functional decline by enhancing muscle contractile capacity and improving motor unit recruitment efficiency (Lake, 1992; Vinck et al., 2006). The pronounced advantage of FES in restoring muscular strength—marked by a >15% improvement in peak concentric power across 24–72 hours—is fundamentally attributed to its unique neural reactivation mechanism. By simultaneously activating spinal reflex pathways and optimizing motor unit recruitment patterns, FES directly addresses the impaired neural drive central to DOMS pathology (Lake, 1992; Vinck et al., 2006). In contrast, while traditional cryotherapy may transiently suppress inflammation, it significantly reduces muscle spindle activity (by up to 30%) and inhibits action potential propagation, thereby exacerbating neuromuscular decoupling and failing to reverse strength loss.

These findings challenge the conventional notion that “inflammation suppression equals recovery” and underscore the priority of neural pathway restoration in effective DOMS rehabilitation.

### 4.2 Effects of different interventions on blood biomarkers associated with DOMS

Regarding changes in IL-6 levels, the results indicated significant main effects of time, group, and their interaction *(p* < 0.01), suggesting that the regulatory effects of different interventions on the inflammatory response differed significantly across time points. IL-6 levels peaked at 24 h post-exercise, coinciding with the exacerbation of DOMS symptoms severity.

The CWI group demonstrated the most pronounced suppression of IL-6 at both 24 and 48 h, effectively controlling the acute inflammatory response and reaffirming the well-established anti-inflammatory benefits of cryotherapy during the early recovery phase. This finding supports the classic view proposed by Peake et al. (2017), which emphasized the strong inhibitory effect of cryotherapy on IL-6 during the early post-exercise window (24–48 h) (Peake et al., 2017). However, for the first time, this study revealed—via temporal monitoring of IL-6 dynamics—that the anti-inflammatory effects of cryotherapy markedly declined by the critical 72-h recovery time point, with IL-6 levels rebounding to 82% of the control group level. This temporal limitation highlights the irreplaceability of active intervention strategies for prolonged recovery phases. However, by 72 h post-exercise, the massage therapy and vibration therapy groups exhibited superior IL-6 reduction, with massage therapy showing the most sustained effect. Notably, this study identified a breakthrough finding: massage therapy produced a 37.5% greater IL-6 reduction than CWI at 72 h post-exercise, confirming the anti-inflammatory pathways proposed by Crane et al. (2012). Furthermore, it elucidated the neurophysiological basis underlying massage’s prolonged anti-inflammatory effect: a dual mechanism involving enhanced peripheral blood flow and activation of the parasympathetic nervous system. These combined effects contribute to systemic inflammatory resolution and effectively alleviate late-phase DOMS symptoms such as muscle soreness and stiffness (Chang et al., 2020; Dupuy et al., 2018; Fridén et al., 1983; Imtiyaz et al., 2014; Karasuno et al., 2016; Tiidus, 1999).

Although the main effect of group and interaction effect for Ca^2+^ concentrations were not statistically significant, the overall trend indicated that the massage therapy group consistently demonstrated superior calcium homeostatic across all time points, outperforming other intervention groups. This suggests that massage therapy may play a positive role in maintaining intracellular and extracellular Ca^2+^ balance, reducing abnormal muscle excitability and spasms, and thereby alleviating DOMS-related muscle stiffness and discomfort (Jaalouk and Lammerding, 2009; Zainuddin et al., 2005). For the first time, this study provided direct temporal evidence supporting the hypothesis proposed by Zainuddin et al. (2005), supporting the hypothesis that massage enhances ATPase (SERCA) activity to stabilize calcium homeostasis (Zainuddin et al., 2005). At all measured time points (24, 48, and 72 h), the massage group exhibited cytosolic free Ca^2+^ fluctuations of less than 15%, significantly lower than those observed in other groups (exceeding 35%). This sustained ability to stabilize intra- and extracellular calcium levels was directly associated with the alleviation of DOMS-related muscle spasms (r = −0.81, p < 0.01). In contrast, both the vibration therapy and static stretching groups showed elevated Ca^2+^ levels during the later stages of recovery, providing empirical support for the caution by Lau and Nosaka. (2011) regarding the risk of calcium overload induced by excessive mechanical stimulation (Lau and Nosaka, 2011). This calcium dysregulation is attributed to sarcolemmal microdamage and mitochondrial Ca^2+^ uptake impairment caused by overstretching, which may prolong excitation–contraction uncoupling in muscle fibers and delay DOMS symptom resolution by 23%–41% (Lau and Nosaka, 2011; Nédélec et al., 2013; Qiu et al., 2022; Toumi and Best, 2003; Wheeler and Jacobson, 2013).

For CK, the analysis revealed statistically significant main effects of both time and group (*p* < 0.05). The period between 24 and 48 h post-exercise represented a critical phase of CK elevation, corresponding to the peak of muscle fiber micro damage associated with DOMS. The massage therapy group showed significantly lower CK levels compared to the control group at 24, 48, and 72 post-exercise hours, with the most effective overall recovery profile. For the first time, this study employed a three-point temporal monitoring approach to confirm that the CK-suppressing effect of massage therapy was not only significant but also sustained, with a cumulative reduction of 38.2%–51.7% across 24–72 h. This finding not only extends the short-term observations of Zainuddin et al. (2005), which were limited to within 24 h, (Zainuddin et al., 2005), but also reveals a dual mechanism: persistent muscle fiber integrity via attenuated membrane permeability (evidenced by a >40% reduction in creatine kinase efflux), and accelerated autophagic clearance of damaged mitochondria (upregulation of LC3-II/Beclin-1). Together, these mechanisms enabled the massage group to become the only intervention to achieve a progressive, stepwise reduction in CK levels throughout the entire recovery period. This finding further supports the pivotal role of massage therapy in reducing muscle damage and promoting tissue repair (Lau and Nosaka, 2011; Wheeler and Jacobson, 2013). Both FES and cold water immersion exhibited moderate benefits at 48 h post-exercise; however, their recovery efficacy remained inferior to those of massage therapy. Notably, the cold-water group exhibited a CK rebound at 72 h (28.3% higher than at 48 h), providing key pathophysiological evidence for the controversial hypothesis proposed by Xiao et al (2023). It is likely that sustained vasoconstriction (>36 h) impaired the clearance of metabolic waste from damaged tissue, thereby prolonging the presence of cellular debris and leading to persistent CK release.

This observation ultimately supports the counter-hypothesis that “anti-inflammatory strategies may potentially delay structural regeneration.” In contrast, CK levels in the control group remained elevated throughout the observation period, indicating that the absence of effective intervention may delay recovery process from DOMS and increase the risk of prolonged muscle soreness and functional impairment.

Nevertheless, it is essential to emphasize that the interventions examined in this study are intended as optional components of DOMS management, rather than mutually exclusive or universally superior solutions. The findings are meant to elucidate differential effects among commonly used strategies—not to advocate for the replacement of one approach with another. In clinical settings, intervention choices should be guided by a combination of factors, including individual preferences, accessibility of resources, and professional guidance.

### 4.3 Limitations of the study

First, the absence of a sham intervention or placebo control group may have limited the ability to control for the substantial placebo effects commonly observed in recovery treatments. Although the findings hold clinical relevance, the lack of placebo-controlled data makes it difficult to clearly distinguish the physiological mechanisms of the interventions from potential psychological modulation effects.

Second, all participants in this study were male. While this design helped control for sex-related confounding variables—such as hormonal fluctuations—thereby allowing clearer interpretation of the physiological effects of the interventions themselves, it also limits the generalizability of the findings to female athletes or broader populations. Future research should incorporate sex-stratified analyses to explore potential sex-based differences in response to DOMS interventions.

Finally, the relatively small sample size per group reduced the statistical power of the study and increased susceptibility to random error, thereby raising concerns about the generalizability of the results. Participants were primarily healthy young male college students, whose physical condition and muscle recovery capacity may exceed that of the general population. This demographic homogeneity may restrict the applicability of the findings. Future studies should aim to expand the sample size and include participants across a broader range of ages, sexes, and health statuses to enhance both external validity and statistical robustness, thereby further substantiating the generalizability of the current findings.

## 5 Conclusion

This study demonstrates that five recovery interventions exhibit targeted advantages in alleviating DOMS. Vibration therapy effectively improved muscle responsiveness and reduced TC by enhancing Type II muscle fiber recruitment and spinal reflex pathways. Massage therapy was the most effective in reducing IL-6, CK, and Ca^2+^ levels, thereby relieving muscle stiffness and promoting tissue repair. FES increased PPT and enhanced muscle strength, thereby mitigating pain and functional decline. Static stretching improved ROM, while cold-water immersion was most effective in controlling early-phase inflammation.

Clinically, given the unique strengths of each intervention, no single modality can comprehensively address all stages of DOMS recovery. We recommend a phase-specific combination strategy involving CWI, massage, stretching, and FES to optimize inflammation control, pain relief, and functional restoration. Specifically, CWI should be prioritized during the acute inflammatory phase (0–24 h) to control initial damage; in the progression phase (24–48 h), massage combined with FES may synergistically regulate pain and tissue repair; during the functional reconstruction phase (48–72 h), stretching integrated with massage can further optimize neuromuscular recovery. This framework establishes a novel paradigm for multi-targeted precision rehabilitation of DOMS, emphasizing stage-specific intervention strategies.

## Data Availability

The raw data supporting the conclusions of this article will be made available by the authors, without undue reservation.
